# Genetic Associations with Non-Syndromic Cleft Lip/Palate and Dental Caries in Kuwaiti Patients: A Case–Control Study

**DOI:** 10.3390/dj14010054

**Published:** 2026-01-13

**Authors:** Manal Abu Al-Melh, Fawzi M. Al-Qatami, Maribasappa Karched, Muawia A. Qudeimat

**Affiliations:** 1 Department of Developmental and Preventive Sciences, College of Dentistry, Kuwait University, Jabriya 13110, Kuwait; muawia.qudeimat@ku.edu.kw; 2 Kuwait Board of Orthodontics & Dentofacial Orthopedics, Kuwait Institute for Medical Specializations, Kuwait City 12050, Kuwait; falqatami@gmail.com; 3 Department of Bioclinical Sciences, College of Dentistry, Kuwait University, Jabriya 13110, Kuwait; maribasappa.karched@ku.edu.kw

**Keywords:** non-syndromic cleft lip and/or palate (NCL/P), dental caries, SNP genotyping, taste receptor polymorphisms, KLK4, TAS1R2

## Abstract

**Background:** Non-syndromic cleft lip/palate (NCL/P) is a prevalent congenital anomaly. Despite an unclear epidemiological link between orofacial clefts and dental caries, genetic studies suggest that polymorphisms in taste receptor genes may influence caries risk. **Objectives:** This study had two primary objectives: (1) to compare SNPs in NCL/P-associated genes (IRF6, FOXE1) between Kuwaiti NCL/P cases and controls, and (2) to explore whether variants in caries-associated (KLK4, DSPP) and taste receptor (TAS1R2, TAS2R38) genes are associated with dental caries susceptibility in individuals with NCL/P, independent of overall caries prevalence. **Methods:** A case–control design was employed, with 25 NCL/P cases and 25 unaffected controls recruited from a Dental Craniofacial Clinic in Kuwait. Genomic DNA was extracted from buccal swabs, and SNP genotyping was performed using real-time PCR for genes related to NCL/P, dental caries, and taste perception. Caries status was assessed using the dmft/DMFT scoring system. The genotyped genes included NCL/P-related (IRF6, FOXE1), caries-related (KLK4, DSPP), and taste receptor genes (TAS1R2, TAS2R38). **Results:** At nominal significance, KLK4, DSPP, and TAS1R2 showed associations with NCL/P status, while IRF6 and FOXE1 did not. After applying Benjamini–Hochberg FDR correction across 10 SNPs, no allele- or genotype-level association remained significant (*q* < 0.05). The strongest signal was KLK4 rs2235091 (allele-level *p* = 0.016; *q* = 0.159). An exploratory age- and sex-adjusted logistic model for KLK4 suggested a possible effect (aOR 0.40; 95% CI 0.18–0.87; *p* = 0.021). Within-group analyses of caries burden revealed no associations that survived FDR control (lowest *q* = 0.056 for FOXE1 in controls). **Conclusions:** After controlling for multiple testing, no SNP showed a statistically significant association with NCL/P or caries burden. Nominal signals for KLK4, DSPP, and TAS1R2 did not survive FDR correction; an exploratory adjusted model suggested a possible KLK4 effect, but this requires cautious interpretation. The small sample size is a key limitation, and the findings highlight the need for larger, well-powered studies to clarify genetic contributions to NCL/P and caries risk.

## 1. Background

Non-syndromic cleft lip and/or palate (NCL/P) is one of the most prevalent congenital anomalies, with reported prevalence rates ranging from 0.5 to 3 per 1000 live births across different populations, genders, and geographic regions [1,2]. In Kuwait, a 2018 study found a prevalence of 0.57 per 1000 live births [3], while a 2024 study reported rates between 0.75 and 2.55 per 1000, varying across different racial groups [4].

The etiology of NCL/P is multifactorial, involving both genetic predispositions and environmental influences, though the exact mechanisms remain incompletely understood [5]. Genetic factors contribute to susceptibility, while environmental triggers such as nutritional deficiencies (e.g., folic acid, vitamin B6, zinc), teratogen exposure, maternal smoking, alcohol use, and high-altitude living interact with susceptible genotypes to influence cleft development [1,6,7]. Approximately 25% of congenital anomalies are attributed to genetic causes, 10% to environmental factors, and 65% to gene-environment interactions [8,9]. Recent studies suggest a polygenic inheritance model for NCL/P, indicating that multiple genes may influence its heritability [10,11].

Among the key genes associated with NCL/P risk, IRF6 (interferon regulatory factor 6) is one of the most studied. Variants of IRF6 are not only associated with NCL/P but also causative in Van der Woude syndrome, the most common syndromic form of cleft lip. IRF6 mutations disrupt epithelial development pathways, leading to cleft malformations [12]. Notably, murine models demonstrate that IRF6 ablation disrupts salivary gland function, reduces saliva buffering capacity, and increases oral colonization by cariogenic bacteria, resulting in a 35-fold higher caries susceptibility [13]. This suggests that IRF6’s role in epithelial integrity may extend beyond cleft etiology to postnatal oral health, potentially explaining reported caries disparities in NCL/P patients. Similarly, FOXE1 (Forkhead Box E1) plays a crucial role in craniofacial development during embryogenesis, and its genetic variations have been linked to NCL/P as well [14,15].

Genetic factors have also been implicated in dental caries risk, with twin studies indicating that 50–85% of caries risk is attributable to genetic background [16,17]. Genome-wide association studies (GWAS) have identified specific polymorphisms in DSPP, KLK4, and AQP5 that influence caries susceptibility. These genes affect dentin composition (DSPP), enamel maturation (KLK4), and salivary function (AQP5), all of which play roles in oral health and caries development [18]. Multiple studies have reported a higher prevalence of caries in individuals with cleft lip and/or palate (CL/P), often attributed to difficulties in oral hygiene, prolonged bottle-feeding, and longer oral clearance times [19,20,21]. However, establishing the precise impact of these risk factors is complicated by the complex genetic and environmental etiology of CL/P itself, as well as the unique genetic susceptibilities across different ethnic groups [5]. Recent evidence suggests that the genetic background of NCL/P may extend beyond the cleft, influencing long-term oral health. For instance, genes like IRF6, crucial for craniofacial development, have also been linked to impaired salivary function and a dramatically increased susceptibility to dental caries [13].

Interestingly, although the connection between orofacial clefts and dental caries remains difficult to establish due to inconsistencies in study designs, research has shown that polymorphisms in taste receptor genes may impact caries risk [16,17,18,22,23,24,25]. These genes influence bitter sensitivity and food preferences, which can indirectly affect dietary choices. For example, individuals with heightened bitter sensitivity tend to avoid bitter-tasting, folate-rich vegetables [26], reducing their folic acid intake, which is a known risk factor for NCL/P in offspring [27]. Additionally, increased bitter sensitivity is associated with a preference for sweet foods, which may lead to higher caries risk due to higher sugar consumption [28,29].

Given these potential overlaps, this study had two primary objectives: (1) to assess whether SNPs in NCL/P-associated genes (IRF6, FOXE1) differ between Kuwaiti NCL/P cases and controls, and (2) to explore whether variants in caries- and taste-related genes (KLK4, DSPP, TAS1R2, TAS2R38) contribute to caries susceptibility in NCL/P patients. By comparing genotype frequencies between NCL/P cases and controls, we aimed to identify genetic markers that could potentially underlie both conditions, offering insights into the observed caries burden in this population.

## 2. Methods

Given murine evidence linking IRF6 to salivary dysfunction and caries [13], we hypothesized that NCL/P patients may carry genetic variants predisposing them to caries. We compared genotype frequencies between NCL/P cases and controls to disentangle cleft-specific from general caries risk factors. The study was approved by the Ethical Committee of the Health Sciences Center, Kuwait University and by the Ethical Committee of the Ministry of Health, Kuwait. Written informed consent was obtained from the legal guardians of each patient participating in the study.

## 3. Sample Selection and Recruitment

The study comprised 25 patients (14 males and 11 females) and 25 controls (12 males and 13 females) recruited from Al-Amiri Dental Craniofacial Clinic, Kuwait. Patients with NCL/P were identified through clinical records, and control participants were recruited based on the absence of craniofacial anomalies. The NCL/P phenotype was determined by thorough clinical examination by a craniofacial orthodontist who examined the presence of cleft palate only (*n* = 2), unilateral cleft lip (*n* = 5), bilateral cleft lip (*n* = 1), unilateral cleft lip and palate (*n* = 9), bilateral cleft lip and palate (*n* = 8).

Participants were evaluated for inclusion/exclusion criteria before their parents provided written informed consent. The study excluded individuals who met any of the following criteria: (i) presence of any systemic condition or craniofacial syndrome, (ii) absence of caries, (iii) diagnosis of salivary gland diseases, and (iv) history of orthodontic treatment. To compare genetic susceptibility while controlling for caries presence, both groups were restricted to participants with active caries (DMFT/dmft > 0). This design allowed us to isolate genetic risk factors from confounding by caries-free status.

## 4. Post Hoc Power Analysis

Post hoc power calculations for KLK4 are provided in Supplementary Table S1 for transparency.

## 5. Dental Examination to Assess Caries

The study participants were examined by two dentists on a dental chair under similar lighting conditions using the WHO criteria for caries examination [30]. A detailed dental exam was performed for each participant, and all findings were recorded. Standard DMFT/dmft (decayed/missing/filled teeth) and DMFS/dmfs (decayed/missing/filled surfaces) scores were measured during the visit when buccal swabs were obtained.

## 6. Sample Collection

Buccal swabs were obtained from all the participants by completely swabbing the inner surface of the cheeks, and the swabs were immersed in 1 mL sterile phosphate-buffered saline (PBS) in a 15 mL sterile tube. The samples were immediately kept on ice after collection, transported to the laboratory, and stored at −80 °C.

## 7. Purification of DNA

Buccal samples stored at −80 °C were thawed and then centrifuged at 10,000× *g* for 10 min as described [31]. In brief, the supernatant was discarded, and the pellet was washed once in 5 mL sterile PBS by centrifuging as above. The final pellet was subjected to DNA purification using the DNeasy Blood & Tissue Kit (QIAGEN, Germantown, MD, USA) according to the manufacturer’s instructions. The concentration of DNA in the purified samples was determined using NanoDrop^TM^ 1000 spectrophotometer, and A260/280 readings were recorded to assess DNA purity.

## 8. SNP Genotyping by Real-Time PCR

In this study, we analyzed three bitter taste receptor SNPs (TAS2R38 rs713598 C/G, TAS2R38 rs1726866 G/A, and TAS2R38 rs10246939 C/T) and two sweet taste receptor SNPs (TAS1R2 rs4920566 A/G and TAS1R2 rs9701796 G/C) to assess their association with NCL/P and dental caries. Additionally, we examined SNPs in NCL/P-related genes (IRF6 rs642961 G/A and FOXE1 rs3758249 G/A) and caries-related genes (DSPP rs2615487 C/T, AQP5 rs1996315 A/G, and KLK4 rs2235091 G/A) to determine their genetic contributions to NCL/P. Custom SNP genotyping assays for the target genes were designed and procured from Applied Biosystems^TM^ (Leicestershire, UK) (Table 1).

SNP genotyping of purified DNA from all clinical samples was performed according to the manufacturer’s instructions. Briefly, for SNP genotyping assays, the reaction mixture included TaqMan Universal PCR Master Mix containing AmpliTaq Gold^®^ (Fisher Scientific, Leicestershire, UK) DNA Polymerase and a working stock of SNP genotyping assay. The custom-made SNP genotyping assay mixture consisted of specific primers labeled with VIC-dye or FAM-dye for the intended SNP targets. The reactions were carried out on a 7500 Fast Real-Time PCR machine using the thermal profile provided by the kit manufacturer. After the completion of the run, an endpoint plate read was performed using the Sequence Detection System (SDS) software to analyze allelic discrimination based on the fluorescence data acquired during the PCR amplification.

## 9. Statistical Analysis

Three independent genotyping experiments were performed for all SNY genotypes. Genetic association analyses were conducted at two levels: (1) case–control comparisons for NCL/P status and (2) within-group analyses of caries burden. For case–control comparisons, allele frequencies were evaluated using two-sided Fisher’s exact tests, and odds ratios (ORs) with 95% confidence intervals (CIs) were calculated, applying the Haldane–Anscombe correction where necessary. Genotype associations were assessed under dominant and recessive models using Fisher’s exact tests. To account for multiple testing, Benjamini–Hochberg false-discovery rate (FDR) correction was applied separately to allele-level tests (m = 10) and to each set of genotype-model tests (m = 10). Exploratory logistic regression models were fitted for SNPs with raw *p* < 0.05, using NCL/P status as the outcome and additive SNP dosage (0/1/2 effect-allele copies) as the predictor, adjusted for age and sex; results are reported as adjusted ORs (aORs) with 95% CIs.

Within-group analyses of caries burden were performed using linear regression of the percentage of caries on additive SNP dosage, adjusted for age and sex, separately in cases and controls; FDR was controlled within each group (m = 10). Hardy–Weinberg equilibrium (HWE) was assessed in controls only. Per-SNP and per-sample call rates were calculated as quality-control metrics. Descriptive statistics for age and caries scores were summarized as means (SD) or medians (IQR) as appropriate. Independent t-tests were used for normally distributed variables (age), and Mann–Whitney U tests for skewed variables (DMFT/dmft percentage). Sex distribution was compared using Fisher’s exact test.

Analyses were performed using Python (SciPy v1.11, StatsModels v0.14, NumFocus, Austin, TX, USA) for genetic and regression analyses and SPSS v29 (IBM Corp., Chicago, IL, USA) for descriptive and non-genetic comparisons. A two-sided α = 0.05 defined nominal significance; FDR *q* < 0.05 defined multiplicity-controlled significance.

## 10. Results

A total of 50 subjects were recruited for the study, comprising 25 cases with NCL/P and 25 unaffected controls. Demographic data, summarized in Table 2, indicated that the cases and controls were matched for age and gender (*p* > 0.05). All participants exhibited varying degrees of dental caries, with a mean caries percentage of 18.2% in cases and 28.5% in controls. However, this difference was not statistically significant (*p* > 0.06).

### 10.1. Genotyping Quality Control

Genotyping quality control is shown in Table 3. The median per-sample call rate was 0.90 (IQR 0.90–1.00). Per-SNP call rates ranged from 0.68 to 1.00. Hardy–Weinberg equilibrium (HWE) was assessed in controls only; IRF6 rs642961 and KLK4 rs2235091 deviated from HWE (see Table 3).

### 10.2. Case–Control Associations

Primary case–control allele-level associations are presented in Table 4. After Benjamini–Hochberg FDR correction across 10 SNPs, no association remained significant at *q* < 0.05. The smallest *q*-value was observed for KLK4 rs2235091 (allele-level Fisher exact *p* = 0.016; *q* = 0.159). An exploratory age- and sex-adjusted logistic model for KLK4 suggested a possible effect (aOR 0.40; 95% CI 0.18–0.87; *p* = 0.021).

### 10.3. Within-Group Analyses of Caries Burden

Within-group analyses of caries burden (percentage of caries) showed no associations that survived FDR control in either cases or controls. The lowest *q*-value was for FOXE1 rs3758249 in controls (β per T allele −16.4%, 95% CI −27.4% to −5.4%; *p* = 0.0056; *q* = 0.056).

## 11. Discussion

NCL/P is a complex congenital anomaly with an increasing incidence among Kuwaiti children in recent years [3,4]. Polymorphisms in several genes involved in orofacial development have been associated with an increased susceptibility to NCL/P [26]. Further, children with NCL/P have shown higher risk for developing dental caries [22,23,24]. A study establishing the association of taste receptor gene polymorphisms and caries risk/protection [25], prompted us to investigate the SNPs of these genes in Kuwaiti children affected with NCL/P, to determine their potential association with caries risk. Additionally, we investigated the association of polymorphisms of NCL/P-related and caries-related genes with NCL/P.

We analyzed SNPs in two NCL/P-related genes, IRF6 and FOXE1. The IRF6 rs642961 G/A SNP showed no significant association with NCL/P in our study, which contrasts with a previous study in an Iranian population that reported an association between IRF6 polymorphisms and NCL/P susceptibility; however, that study analyzed different IRF6 SNPs (rs2013162 and rs2235375) [32]. Similarly, the FOXE1 rs3758249 C/T SNP showed no significant association with NCL/P, aligning with findings from a Kuwaiti population study [33]. However, this result contradicts studies in Northeast Chinese, Brazilian, and European populations, which reported a strong association between FOXE1 rs3758249 and NCL/P [14,15,34,35,36,37]. These discrepancies may be due to ethnic genetic diversity, differences in sample sizes, or the specific oral cleft subtypes analyzed. Additionally, other SNPs such as LOC102724968 rs13041247, PVT1 rs987525, CYRIA rs7552, MSX1 rs3821949, and TGFB3 rs3917201 have been associated with NCL/P in the Kuwaiti population [4,33]. Further investigation of additional SNPs in the IRF6 and FOXE1 genes is necessary before definitively ruling out their role in NCL/P susceptibility.

The correlation between orofacial clefts and dental caries has been difficult to confirm due to inconsistencies in study designs, which involve various variables such as cleft type, the inclusion of syndromes, subject ages, and the quality of preventive care [38,39,40,41,42]. Supporting an association between NCL/P and caries, one study comparing children with NCL/P to their cleft-free siblings found a higher prevalence of dental caries in those with NCL/P, as reflected by higher DMFT index scores, suggesting increased susceptibility to caries regardless of socioeconomic status [42]. In contrast, another study found no significant difference in caries frequency between children with NCL/P and control children [39]. A more recent study in England, which examined hospital admissions for dental treatment among children with NCL/P born between 1997 and 2003, found that 66% of admissions were primarily for caries, with 96% of these cases involving extractions [40]. A systematic review of studies on dental caries in children with CLP found a tendency toward higher caries scores in preschool children with CLP. However, due to conflicting results, the evidence remained inconclusive about whether children with CLP have more caries than non-cleft controls. The quality of the studies included in the review was rated as low to moderate, and the results were inconsistent [38]. As a result, the authors concluded that it is crucial to reduce hospital admissions and emphasize prevention, especially for individuals at high risk for caries. Preventive dental care is particularly important for patients with CL/P compared to those without clefts [41]. However, there is still a lack of research on the potential genetic factors underlying this association. Research indicates that both environmental factors (such as age, tooth-brushing frequency, and water fluoride levels) and genetic factors (including polymorphisms in the KLK4, DSPP, and AQP5 genes) contribute to the progression of dental caries [18]. Wang et al. (2012) reported a significant association between the A allele of the KLK4 rs2235091 SNP and the T allele of the DSPP rs2615487 SNP, both of which were linked to protection against dental caries [18]. Conversely, Gachova et al. (2023) found that the A allele of KLK4 rs2235091 SNP was associated with an increased risk of dental caries [43]. These discrepancies highlight the ongoing ambiguity in genetic associations with dental caries. A systematic review and meta-analysis concluded that the KLK4 rs2236091 SNP may be associated with caries in primary dentition but not permanent dentition [44]. In our study, the KLK4 G/A SNP showed significant differences in both genotype distribution and allele frequency between groups, with NCL/P cases exhibiting higher A allele prevalence. In contrast, the DSPP C/T SNP demonstrated only marginal allele frequency differences without significant genotype distribution variations. The Hardy–Weinberg equilibrium consistency and lack of strong associations suggest this SNP’s role in caries susceptibility may be limited for NCL/P individuals, though further investigation is warranted. The coexistence of enriched caries-risk alleles (KLK4, DSPP) in NCL/P patients with comparable caries prevalence to controls suggests these individuals may require sustained preventive care to counteract inherent genetic susceptibility. The absence of elevated caries scores in NCL/P cases likely reflects the effectiveness of rigorous hygiene protocols in this population. However, the precise relationship between these polymorphisms and caries risk in Kuwaiti NCL/P children requires clarification through additional research.

A previous study evaluated genetic variation in taste pathway genes, specifically TAS2R38 and TAS1R2, and their association with caries susceptibility in an Appalachian population [25]. Genotyping assays were performed on nearly 2500 individuals, followed by dental examinations to assess caries. The analysis revealed a significant association between specific TAS2R38 alleles and protection against dental caries [25]. In the current study, we investigated the potential role of TAS2R38 and TAS1R2 taste receptor genes in the susceptibility to NCL/P and dental caries. Overall, our results suggest that these genes are not strongly associated with NCL/P or dental caries in the study population. Although TAS1R2 G/C exhibited a borderline significant result for genotype distribution and a marginal trend in allele frequencies, the lack of consistent significant findings across other SNPs and tables indicates that TAS2R38 and TAS1R2 are not major contributors to the studied conditions. Additionally, Hardy–Weinberg equilibrium analysis for TAS1R2 G/C in the case group revealed a slight deviation, which may indicate a selective force or a population-specific genetic distribution. However, this does not necessarily suggest a direct role in NCL/P or caries susceptibility. These findings highlight the need for further studies with larger sample sizes and diverse populations to fully explore the potential genetic influences of taste receptors on NCL/P and dental caries, as well as the possibility of more subtle or indirect effects. Our findings contrast with a previous study investigating the same taste receptor SNPs in NCL/P patients from India, which found no significant genotype associations for any of the five variants. However, that study observed a significantly lower frequency of protective alleles against dental caries for three variants (TAS2R38 rs713598, TAS2R38 rs10246939, and TAS1R2 rs4920566) in NCL/P cases compared to controls [45]. Furthermore, a recent systematic review and meta-analysis found that children carrying the rs9701796 variant exhibited dietary patterns associated with increased consumption of sweet foods, which is linked to a higher risk for dental caries [46].

Patients with cleft lip and/or palate (CL/P) present with a complex congenital craniofacial anomaly that necessitates multiple staged surgical interventions to achieve satisfactory functional and esthetic outcomes. A substantial proportion of patients (25–60%) with complete unilateral CL/P require maxillary advancement to correct midfacial retrusion and reestablish facial balance [47,48,49,50]. Since several decades, orthognathic surgery has represented the conventional approach for addressing skeletal deformities associated with CL/P. Compared to individuals without clefts, patients with CL/P frequently exhibit midfacial and maxillary hypoplasia, warranting surgical advancement for both functional rehabilitation and esthetic refinement. Attaining optimal surgical and esthetic results in orthognathic surgery among CL/P patients is inherently more demanding than in noncleft counterparts. Common challenges include extensive scarring from prior soft-tissue procedures and the requirement for substantial maxillary advancement. However, despite these complexities, existing literature indicates that orthognathic surgery in CL/P patients does not necessarily present a higher risk of postoperative complications compared to orthognathic surgery in patients without clefts [51,52].

This study has several limitations. First, the small sample size, though justified by the rarity of NCL/P in Kuwait, limits generalizability. While the study suggests biological relevance, replication in larger cohorts is needed to confirm the observed nominal associations. Second, by excluding caries-free participants, we prioritized the study of genetic risk in caries-active individuals but could not assess whether these variants also predict caries incidence. Third, despite adjusting for age and sex, unmeasured confounders (e.g., dietary habits, oral hygiene practices, or socioeconomic factors) may influence caries risk. We did not collect data on preventive care intensity, fluoridation exposure, tooth-brushing habits, diet, or socioeconomic status; this is a key limitation and residual confounding is likely. Fourth, deviations from Hardy–Weinberg equilibrium for *KLK4* and *TAS1R2* SNPs in NCL/P cases may reflect selection bias, population stratification, or technical artifacts, warranting cautious interpretation. The lack of consistent associations for taste receptor genes suggests their limited role in caries susceptibility in this cohort, though subtle effects cannot be ruled out. Finally, the pooling of different NCL/P subtypes may obscure potential subtype-specific genetic effects, and the lack of consistent associations for taste receptor genes suggests their limited role in caries susceptibility in this cohort, though subtle effects cannot be ruled out. These limitations underscore the need for a larger study with comprehensive phenotyping to validate our findings. Future directions should include larger, multi-ethnic studies with genome-wide approaches, detailed phenotyping of oral hygiene and dietary habits, and analysis of gene-environment interactions to fully elucidate the genetic architecture underlying caries risk in NCL/P patients.

## 12. Conclusions

Although no statistically significant associations were observed between NCL/P and IRF6 or FOXE1 variants, exploratory findings suggest a potential link between KLK4 and caries susceptibility in individuals with NCL/P. Variants in taste receptor genes such as TAS1R2 showed inconsistent signals and did not remain significant after correction for multiple testing. The absence of significant differences in caries experience between groups may reflect the impact of enhanced preventive care among NCL/P patients, potentially masking underlying genetic risk. These findings highlight the need for larger, multi-ethnic studies with comprehensive environmental and behavioral data to clarify genetic contributions to caries risk in this population.

## Figures and Tables

**Table 1:** List of target gene polymorphisms studied.

Gene	SNP
NCL/P-Related	IRF6 rs642961 G/A
	FOXE1 rs3758249 C/T
Caries-Related	DSPP rs2615487 C/T
	AQP5 (rs1996315) A/G
	KLK4 rs2235091 G/A
Taste Receptor	TAS2R38 rs713598 C/G
	TAS2R38 rs1726866 G/A
	TAS2R38 rs10246939 C/T
	TAS1R2 rs4920566 A/G
	TAS1R2 rs9701796 G/C

**Table 2:** Demographic characteristics of NCL/P patients compared to those of control.

Variable		NCL/P Cases	Control	*p*-Value
Sample Size	─	25	25	─
Age in years	Mean (SD)	6.5 (3.3)	7.6 (1.8)	0.17
	Range	2.5–14.8	3.7–10.8	
Sex	Male (%)	14 (56)	12 (48)	0.32
	Female (%)	11 (44)	13 (52)	
Normalized DMFT/dmft % (SD)	─	18.24 (18.5)	28.52 (17.7)	0.06

**Table 3:** Genotyping quality control: per-SNP call rates and control-only Hardy–Weinberg equilibrium (HWE) of genes associated with NCL/P, dental caries, and taste receptors among patients with NCL/P and controls.

Genes	SNP	Alleles	Call Rate (All)	Call Rate (Cases)	Call Rate (Controls)	Control HWE *p*
	IRF6 rs642961 G/A	G/A	0.98	1.0	0.96	0.0015
NCL/P-Related	FoxE1 rs3758249 C/T	C/T	0.9	0.92	0.88	0.67
	DSPP rs2615487 C/T	C/T	0.74	0.76	0.72	0.35
Caries-Related	AQP5 rs1996315 A/G	A/G	0.98	0.96	1.0	0.74
	KLK4 rs2235091 G/A	G/A	1.0	1.0	1.0	0.017
	TAS2R38 rs713598 C/G	C/G	0.98	0.96	1.0	0.74
Taste Receptor	TAS2R38 rs1726866 G/A	G/A	0.98	1.0	0.96	0.74
	TAS2R38 rs10246939 C/T	C/T	1.0	1.0	1.0	0.90
	TAS1R2 rs4920566 A/G	A/G	1.0	1.0	1.0	1.0
	TAS1R2 rs9701796 G/C	G/C	0.68	0.56	0.8	0.52

**Table 4:** Case–control allele-level associations: allele counts (effect/other), odds ratios (OR) with 95% CIs, exact two-sided *p*-values, and FDR *q*-values of genes associated with NCL/P, dental caries, and taste receptors among patients with NCL/P and controls.

Genes	SNP	Effect Allele	Other Allele	Cases (Effect/Other)	Controls (Effect/Other)	OR	95% CI (L)	95% CI (U)	*P* (Fisher Exact)	FDR *q*
NCL/P-Related	IRF6 rs642961 G/A	A	G	45/5	37/11	2.68	0.85	8.39	0.10	0.181
	FoxE1 rs3758249 C/T	T	C	16/30	11/33	1.6	0.64	3.99	0.36	0.453
Caries-Related	DSPP rs2615487 C/T	T	C	11/27	19/17	0.36	0.14	0.95	0.057	0.181
	AQP5 rs1996315 A/G	G	A	25/23	31/19	0.67	0.3	1.49	0.41	0.461
	KLK4 rs2235091 G/A	A	G	19/31	32/18	0.34	0.15	0.78	0.015	0.159
Taste Receptor	TAS2R38 rs713598 C/G	G	C	27/21	21/29	1.78	0.8	3.95	0.22	0.322
	TAS2R38 rs1726866 G/A	G	A	26/24	30/20	0.72	0.33	1.6	0.56	0.546
	TAS2R38 rs10246939 C/T	T	C	19/31	28/22	0.48	0.22	1.07	0.11	0.181
	TAS1R2 rs4920566 A/G	A	G	31/19	21/27	2.1	0.94	4.7	0.10	0.181
	TAS1R2 rs9701796 G/C	C	G	9/19	5/35	3.32	0.97	11.32	0.07	0.181

## Data Availability

The datasets used and/or analyzed during the current study are available from the corresponding author on reasonable request.

## Supplementary Materials

The following supporting information can be downloaded at: https://www.mdpi.com/article/10.3390/dj14010054/s1. Table S1: Post Hoc Power Calculations.

## Abbreviations

SNPs: single nucleotide polymorphisms; NCL/P: non-syndromic cleft lip and/or palate; dmft/DMFT: decayed/missing/filled teeth; dmfs/DMFS: decayed/missing/filled surface; KLK4: kallikrein-related peptidase 4; AQP5: aquaporin 5; PBS: phosphate-buffered saline. HWE: Hardy–Weinberg Equilibrium.

## References

[1] Murray J.C. (1995). Face facts: Genes, environment, and clefts. Am. J. Hum. Genet..

[2] Tolarova M.M., Cervenka J. (1998). Classification and birth prevalence of orofacial clefts. Am. J. Med. Genet..

[3] Alhayyan W.A., Pan S.C., AlQatami F.M. (2018). Birth Prevalence of Orofacial Clefts in Kuwait from Hospital-Based Registration: Retrospective Study. Cleft Palate Craniofac. J..

[4] Alkharafi L., Mokhtar A., Burezq H., Almerjan D., Dashti G., Almutalaqem R., Alshammari A., Alhasawi S., Alqatami F., Geevarghese A. (2024). Seasonal, Geographic, and Ethnic Influence on the Prevalence of Orofacial Clefts in Kuwait: A Nationwide Study. Cleft Palate Craniofac. J..

[5] Dixon M.J., Marazita M.L., Beaty T.H., Murray J.C. (2011). Cleft lip and palate: Understanding genetic and environmental influences. Nat. Rev. Genet..

[6] Tolarova M.M.T., Porter M., Olson C., Tinloy J., Ku L., Louie A., Abouchon A., Pawar T., Obara M., Wu J. Candidate genes and candidate nutrients in etiology of orofacial clefts. Proceedings of the 3rd International Workshop of International Cleft Lip and Palate Foundation CLEFT2011.

[7] Nelson K., Holmes L.B. (1989). Malformations due to presumed spontaneous mutations in newborn infants. N. Engl. J. Med..

[8] Brent R.L. (2004). Environmental causes of human congenital malformations: The pediatrician’s role in dealing with these complex clinical problems caused by a multiplicity of environmental and genetic factors. Pediatrics.

[9] Murray J.C. (2002). Gene/environment causes of cleft lip and/or palate. Clin. Genet..

[10] Wyszynski D.F., Beaty T.H., Maestri N.E. (1996). Genetics of nonsyndromic oral clefts revisited. Cleft Palate-Craniofac. J..

[11] Leslie E.J., Marazita M.L. (2013). Genetics of cleft lip and cleft palate. Am. J. Med. Genet. C Semin. Med. Genet..

[12] Zucchero T.M., Cooper M.E., Maher B.S., Daack-Hirsch S., Nepomuceno B., Ribeiro L., Caprau D., Christensen K., Suzuki Y., Machida J. (2004). Interferon regulatory factor 6 (IRF6) gene variants and the risk of isolated cleft lip or palate. N. Engl. J. Med..

[13] Tamasas B., Cox T.C. (2017). Massively Increased Caries Susceptibility in an Irf6 Cleft Lip/Palate Model. J. Dent. Res..

[14] Marazita M.L., Lidral A.C., Murray J.C., Field L.L., Maher B.S., Goldstein McHenry T., Cooper M.E., Govil M., Daack-Hirsch S., Riley B. (2009). Genome scan, fine-mapping, and candidate gene analysis of non-syndromic cleft lip with or without cleft palate reveals phenotype-specific differences in linkage and association results. Hum. Hered..

[15] Moreno L.M., Mansilla M.A., Bullard S.A., Cooper M.E., Busch T.D., Machida J., Johnson M.K., Brauer D., Krahn K., Daack-Hirsch S. (2009). FOXE1 association with both isolated cleft lip with or without cleft palate, and isolated cleft palate. Hum. Mol. Genet..

[16] Bretz W.A., Corby P.M., Melo M.R., Coelho M.Q., Costa S.M., Robinson M., Schork N.J., Drewnowski A., Hart T.C. (2006). Heritability estimates for dental caries and sucrose sweetness preference. Arch. Oral Biol..

[17] Wang X., Shaffer J.R., Weyant R.J., Cuenco K.T., DeSensi R.S., Crout R., McNeil D.W., Marazita M.L. (2010). Genes and their effects on dental caries may differ between primary and permanent dentitions. Caries Res..

[18] Wang X., Willing M.C., Marazita M.L., Wendell S., Warren J.J., Broffitt B., Smith B., Busch T., Lidral A.C., Levy S.M. (2012). Genetic and environmental factors associated with dental caries in children: The Iowa Fluoride Study. Caries Res..

[19] Antonarakis G.S., Palaska P.K., Herzog G. (2013). Caries prevalence in non-syndromic patients with cleft lip and/or palate: A meta-analysis. Caries Res..

[20] Shashni R., Goyal A., Gauba K., Utreja A.K., Ray P., Jena A.K. (2015). Comparison of risk indicators of dental caries in children with and without cleft lip and palate deformities. Contemp. Clin. Dent..

[21] Sundell A.L., Ullbro C., Marcusson A., Twetman S. (2015). Comparing caries risk profiles between 5- and 10-year-old children with cleft lip and/or palate and non-cleft controls. BMC Oral Health.

[22] Britton K.F., Welbury R.R. (2010). Dental caries prevalence in children with cleft lip/palate aged between 6 months and 6 years in the West of Scotland. Eur. Arch. Paediatr. Dent..

[23] Bokhout B., Hofman F.X., van Limbeek J., Kramer G.J., Prahl-Andersen B. (1996). Increased caries prevalence in 2.5-year-old children with cleft lip and/or palate. Eur. J. Oral Sci..

[24] Bokhout B., Hofman F.X., van Limbeek J., Kramer G.J., Prahl-Andersen B. (1997). Incidence of dental caries in the primary dentition in children with a cleft lip and/or palate. Caries Res..

[25] Wendell S., Wang X., Brown M., Cooper M.E., DeSensi R.S., Weyant R.J., Crout R., McNeil D.W., Marazita M.L. (2010). Taste genes associated with dental caries. J. Dent. Res..

[26] Drewnowski A., Gomez-Carneros C. (2000). Bitter taste, phytonutrients, and the consumer: A review. Am. J. Clin. Nutr..

[27] Badovinac R.L., Werler M.M., Williams P.L., Kelsey K.T., Hayes C. (2007). Folic acid-containing supplement consumption during pregnancy and risk for oral clefts: A meta-analysis. Birth Defects Res. A Clin. Mol. Teratol..

[28] Keller K.L., Tepper B.J. (2004). Inherited taste sensitivity to 6-n-propylthiouracil in diet and body weight in children. Obes. Res..

[29] World Health Organization (2013). Oral Health Surveys Basic Methods.

[30] Qudeimat M.A., Alyahya A., Karched M., Behbehani J., Salako N.O. (2021). Dental plaque microbiota profiles of children with caries-free and caries-active dentition. J. Dent..

[31] Khan A.N.M.I., Prashanth C.S., Srinath N. (2020). Genetic etiology of cleft lip and palate. AIMS Mol. Sci..

[32] Soleymani M., Ebadifar A., Khosravi M., Esmaeilzadeh E., Khorram Khorshid H.R. (2022). Association of rs2013162 and rs2235375 Polymorphisms in IRF6 Gene with Susceptibility to Non-Syndromic Cleft Lip and Palate. Avicenna J. Med. Biotechnol..

[33] Abdelhafez N., Aladsani A., Alkharafi L., Al-Bustan S. (2025). Association of selected gene variants with nonsyndromic orofacial clefts in Kuwait. Gene.

[34] Liu K., Lu Y., Ai L., Jiao B., Yu J., Zhang B., Liu Q. (2015). Association between FOXE1 and non-syndromic orofacial clefts in a northeastern Chinese population. Br. J. Oral Maxillofac. Surg..

[35] do Rego Borges A., Sa J., Hoshi R., Viena C.S., Mariano L.C., de Castro Veiga P., Medrado A.P., Machado R.A., de Aquino S.N., Messetti A.C. (2015). Genetic risk factors for nonsyndromic cleft lip with or without cleft palate in a Brazilian population with high African ancestry. Am. J. Med. Genet. A.

[36] Jugessur A., Shi M., Gjessing H.K., Lie R.T., Wilcox A.J., Weinberg C.R., Christensen K., Boyles A.L., Daack-Hirsch S., Trung T.N. (2009). Genetic determinants of facial clefting: Analysis of 357 candidate genes using two national cleft studies from Scandinavia. PLoS ONE.

[37] Venza M., Visalli M., Venza I., Torino C., Tripodo B., Melita R., Teti D. (2009). Altered binding of MYF-5 to FOXE1 promoter in non-syndromic and CHARGE-associated cleft palate. J. Oral Pathol. Med..

[38] Hasslof P., Twetman S. (2007). Caries prevalence in children with cleft lip and palate—A systematic review of case-control studies. Int. J. Paediatr. Dent..

[39] Tannure P.N., Costa Mde C., Kuchler E.C., Romanos H.F., Granjeiro J.M., Vieira A.R. (2012). Caries experience in individuals with cleft lip and palate. Pediatr. Dent..

[40] Fitzsimons K.J., Copley L.P., Smallridge J.A., Clark V.J., van der Meulen J.H., Deacon S.A. (2014). Hospital admissions for dental treatment among children with cleft lip and/or palate born between 1997 and 2003: An analysis of Hospital Episode Statistics in England. Int. J. Paediatr. Dent..

[41] Cheng L.L., Moor S.L., Ho C.T. (2007). Predisposing factors to dental caries in children with cleft lip and palate: A review and strategies for early prevention. Cleft Palate Craniofac. J..

[42] Al-Dajani M. (2009). Comparison of dental caries prevalence in patients with cleft lip and/or palate and their sibling controls. Cleft Palate Craniofac. J..

[43] Gachova D., Lipovy B., Deissova T., Izakovicova Holla L., Danek Z., Borilova Linhartova P. (2023). Polymorphisms in genes expressed during amelogenesis and their association with dental caries: A case-control study. Clin. Oral Investig..

[44] Li Y., Zhang L., Cen W., Yuan Y. (2023). Association of KLK4 rs2235091 polymorphism with susceptibility to dental caries: A systematic review and meta-analysis. Front. Pediatr..

[45] Courtney Ray R.L.B., Marie M. (2011). Tolarova: Polymorphisms of Taste Receptor Genes Associated with Dental Caries and Cleft Lip and Palate Anomalies in Udaipur, India.

[46] Motahari P., Molaei Z., Adhami Z.E. (2024). Association of TAS1R2 (rs35874116 or rs9701796) Gene Polymorphism with Dental Caries: A Systematic Review and Meta-analysis. Open Dent. J..

[47] Daskalogiannakis J., Mehta M. (2009). The need for orthognathic surgery in patients with repaired complete unilateral and complete bilateral cleft lip and palate. Cleft Palate-Craniofac. J..

[48] Panula K., Lovius B.B., Pospisil O.A. (1993). The need for orthognathic surgery in patients born with complete cleft palate or complete unilateral cleft lip and palate. Oral Surg. Oral Diagn..

[49] Rachmiel A. (2007). Treatment of maxillary cleft palate: Distraction osteogenesis versus orthognathic surgery—Part one: Maxillary distraction. J. Oral Maxillofac. Surg..

[50] Ross R.B. (1987). Treatment variables affecting facial growth in complete unilateral cleft lip and palate. Cleft Palate-Craniofac. J..

[51] Cheung L.K., Chua H.D. (2006). A meta-analysis of cleft maxillary osteotomy and distraction osteogenesis. Int. J. Oral Maxillofac. Surg..

[52] Jedrzejewski M., Smektala T., Sporniak-Tutak K., Olszewski R. (2015). Preoperative, intraoperative, and postoperative complications in orthognathic surgery: A systematic review. Clin. Oral Investig..

